# EEG Effective Source Projections Are More Bilaterally Symmetric in Infants Than in Adults

**DOI:** 10.3389/fnhum.2020.00082

**Published:** 2020-03-12

**Authors:** Caterina Piazza, Chiara Cantiani, Makoto Miyakoshi, Valentina Riva, Massimo Molteni, Gianluigi Reni, Scott Makeig

**Affiliations:** ^1^Scientific Institute, IRCCS Eugenio Medea, Bioengineering Lab, Lecco, Italy; ^2^Scientific Institute, IRCCS Eugenio Medea, Child Psychopathology Unit, Lecco, Italy; ^3^Swartz Center for Computational Neuroscience, Institute for Neural Computation, University of California, San Diego, La Jolla, CA, United States

**Keywords:** EEG, independent component analysis, functional brain organization, development, brain laterality, brain symmetry

## Abstract

Although anatomical brain hemispheric asymmetries have been clearly documented in the infant brain, findings concerning functional hemispheric specialization have been inconsistent. The present report aims to assess whether bilaterally symmetric synchronous activity between the two hemispheres is a characteristic of the infant brain. To asses cortical bilateral synchronicity, we used decomposition by independent component analysis (ICA) of high-density electroencephalographic (EEG) data collected in an auditory passive oddball paradigm. Decompositions of concatenated *64-channel* EEG data epochs from each of 34 typically developing 6-month-old infants and from 18 healthy young adults participating in the same passive auditory oddball protocol were compared to characterize differences in functional brain organization between early life and adulthood. Our results show that infant EEG decompositions comprised a larger number of independent component (IC) effective source processes compatible with a cortical origin and having bilaterally near-symmetric scalp projections (13.8% of the infant data ICs presented a bilateral pattern vs. 4.3% of the adult data ICs). These IC projections could be modeled as the sum of potentials volume-conducted to the scalp from synchronous locally coherent field activities in corresponding left and right cortical source areas. To conclude, in this paradigm, source-resolved infant brain EEG exhibited more bilateral synchronicity than EEG produced by the adult brain, supporting the hypothesis that more strongly unilateral and likely more functionally specialized unihemispheric cortical field activities are concomitants of brain maturation.

## Introduction

The existence of structural and functional hemispheric asymmetries supporting differential specialization of the left and right hemispheres has been widely documented in the adult brain (Toga and Thompson, 2003). This hemispheric specialization supports execution of cognitive, behavioral, emotional, and motor functions and deficiencies in this brain asymmetry are suspected to be involved in various neurodevelopmental disorders (e.g., autism spectrum disorder and language disorders; Herbert et al., 2005; Bishop, 2013). Thus, much effort has been devoted to characterize the development of brain lateralization in early life stages at both structural and functional levels. Brain magnetic resonance imaging (MRI) studies have shown that some structural asymmetry is already present in early infancy (Li et al., 2014; Dean et al., 2018). However, anatomical asymmetry does not necessarily entail functional asymmetry, and reports of functional hemispheric specialization in infancy have been mixed. Some studies have supported the hypothesis that early structural asymmetry is associated with functional hemispheric asymmetry, and have identified asymmetric sensory activation patterns similar to those identified in adults, but overall these reports have been inconsistent.

For example, while the fMRI study of Dehaene-Lambertz et al. (2002) reported a left lateralized BOLD activation associated with speech stimuli in 3-month-old infants, Ortiz-Mantilla et al. (2012) did not identify a similar lateralization effect in electroencephalographic (EEG) source-resolved evoked responses to speech-related stimuli in 6-month-old infants. Further, Telkemeyer et al. (2009, 2011) studied hemodynamic [functional near-infrared spectroscopy (fNIRS)] responses to temporally structured non-speech stimuli, reporting a dominant right-hemisphere activation in the processing of slow temporal modulations in neonates, 3-month and 6-month old infants. Whereas, Minagawa-Kawai et al. (2011) reported bilateral hemodynamic fNIRS activation in response to both rapidly and slowly temporally modulated sounds in newborns. EEG results of Musacchia et al. (2013) suggested an overall greater involvement of the right hemisphere in processing of tones in 4-month-old infants, but a left-lateralized response to rapid pitch changes. This latter result has been recently confirmed in 6-month-old typically developing infants, whereas infants at high risk for language impairment demonstrated a more right-lateralized pattern (Cantiani et al., 2019). Homae (2014), in his review of literature on the development of interhemispheric organization using fNIRS measures, summarized studies showing both bilateral and unilateral activation patterns in infant populations.

In our previous EEG study (Piazza et al., 2016b) of the cortical correlates of multi-feature rapid auditory processing in 6-month-old infants, decomposition by independent component analysis (ICA) of the whole EEG records identified the presence of a relatively large number of near bilaterally symmetric independent component (IC) processes indicating a bilaterally synchronous cortical activity (Piazza et al., 2016a). The present report aims to better investigate bilateral synchronicity by comparing infant and adult EEG data recorded using the same protocol, to assess whether brain EEG activity in infants may indeed exhibit more bilateral synchronicity at the cortical effective source level than in the adult brain.

Independent component analysis decomposition is particularly suitable for this purpose. As a “blind separation” method, it separates the data into spatially static and maximally temporally independent activities, without any consideration as to the physiological nature of their sources. In most cases, ICA isolates the potentials projecting from a single cortical “effective source” area within which local field activity is coherent in full or part, thus returning ICs with (single) dipolar scalp projections. In other cases, much less frequently, ICA returns single ICs with bilaterally distributed scalp projections from two non-adjacent, near-symmetrically located equivalent current dipoles. In these cases, the source process modeled by each IC can be well modeled by the sum, at the scalp electrodes, of locally coherent field activities projecting synchronously to the scalp not from one but from two, non-adjacent left and right, and thus physiologically coupled, effective source areas (Piazza et al., 2016a). A single IC source process associated with a bilateral scalp projection pattern should provide stronger evidence of bilateral synchronicity than that observed in, e.g., scalp-channel event-related potential (ERP) peaks or BOLD signal correlations. Indeed, in order to escape being assigned to different ICs, left and right source activities must exhibit, throughout the data, a consistent left/right amplitude ratio and relative phase at involved frequencies. The bilaterality of the source projections is not much influenced by individual differences in head size and shape, making ICA decomposition an appropriate method for comparing adult and infant EEG data. Moreover, ICA has been frequently used to investigate functional connectivity in fMRI studies (e.g., Joel et al., 2011; Wu et al., 2018).

## Materials and Methods

### Participants

Thirty-four typically developing infants (16 males and 18 females; age in months M = 6.4, SD = 0.4) were included in the present study. The infant group is the same that took part in the study presented in Piazza et al. (2016b). Infants were included in the study if: (1) both parents were native-Italian speakers, (2) gestational age was ≥ 37 weeks, (3) birth-weight was ≥ 2500 g, (4) APGAR scores at birth at 1’and at 5’were ≥ 8, (5) infants did not have a history of hearing impairments and passed the hearing screening at birth, (6) infants and their first-degree relatives did not have certified diagnosis of neurodevelopmental or neurological disorders, and (7) the cognitive subscale of the Bayley Scales of Infant Development (Bayley, 1993) was ≥ 7.

Twenty-one healthy young adults were enrolled. The adult subjects were included in the study if: (1) were native-Italian speakers, (2) did not report any hearing impairments, and (3) did not have certified diagnosis of neurodevelopmental or neurological disorders. Data from 18 adults (nine males and nine females; mean age in years M = 24.7, SD = 4.1) were analyzed; data of three subjects were excluded for having an insufficient amount of usable EEG data. Among the included subjects 16 were right-handed, one was left-handed, and one ambidextrous.

The study was approved by the Ethics Committee of the Scientific Institute IRCCS E. Medea (Bosisio Parini, LC, Italy); written informed consent to participate was obtained from all adult subjects and infant’s parent or guardian prior to inclusion in the protocol.

### EEG Data Recording

Electroencephalographic signals were recorded using a dense-array EGI system (Electric Geodesic, In., Eugene, OR, United States). Adult caps comprised 64 channels, infant caps 60 channels. During collection, EEG data were referenced online to Cz, sampled at 250 Hz, and bandpass filtered between 0.1 and 100 Hz.

**FIGURE 1:**
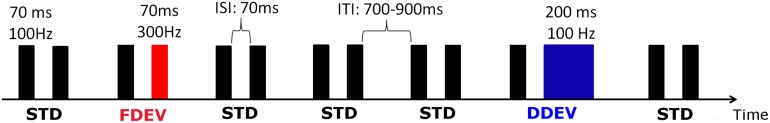
Schematic representation of the auditory oddball paradigm. STD stimuli (black) were composed of two identical tones [F0 = 100 Hz, duration = 70 ms, inter-stimulus interval (ISI) 70 ms], whereas in the deviant stimuli (DEV), the second tone had a fundamental frequency of 300 Hz (FDEV, in red) or it lasted 200 ms (DDEV, in blue). Inter-trial interval (ITI) varied randomly from 700 to 900 ms. In each session, 1200 stimuli (80% STD, 10% FDEV, 10% DDEV) were delivered in pseudo-random order with the constraint of at least three STD stimulus presentations between DEV pairs.

Auditory ERPs were recorded using the non-speech, multi-feature auditory oddball paradigm presented in Piazza et al. (2016b), in which frequent “standard” tone-pairs (STD) were interspersed with occasional frequency or duration “deviant” tone-pairs (FDEV and DDEV, respectively) (Figure 1). In both the adult and infant protocols, participant attention was directed toward an age appropriated silent movie/cartoon. Infant vigilance status was evaluated by the experimenter who gave a score (from 1 = sleeping to 5 = very alert) both at the beginning and at the end of the EEG recording. No statistical differences were found between the initial and the final vigilance status (paired *t*-test, *p* = 0.615). The mean score assigned was 3.19 (SD = 0.68), associated in this scale with a quite alert status.

### EEG Data Processing

**FIGURE 2:**
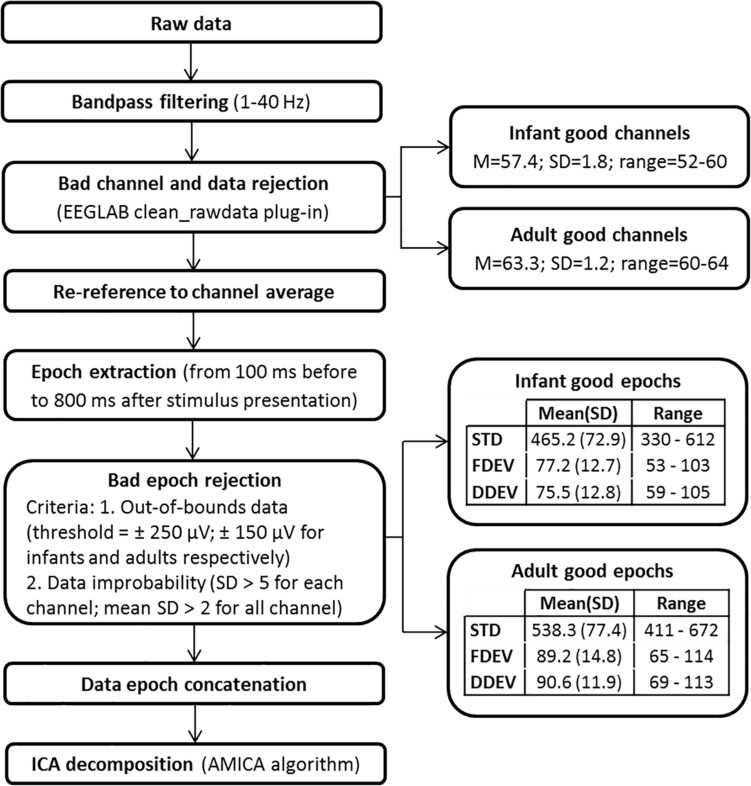
Data preprocessing pipeline.

Electroencephalographic data were exported in a Matlab-compatible format (The Mathworks, Natick, MA, United States) and processed within the open source EEGLAB signal processing environment (Delorme and Makeig, 2004). The ICA decomposition pipeline, shown schematically in Figure 2, is the same used in Piazza et al. (2016b). The continuous raw EEG data were filtered with a symmetric 1-Hz high-pass and then 40-Hz low-pass FIR filters. The *clean_rawdata* EEGLAB plug-in was used to remove flat channels (i.e., channels with flat line duration > 5 s) and channels poorly correlated with their interpolated reconstruction based on neighboring channels (correlation threshold = 0.85). The same plug-in was used to reject data periods in which more than 50% of the channels were contaminated by artifact. The EEG signals were then re-referenced to average reference and segmented in 900-ms epochs. The POz channel was discarded to compensate for the data rank reduction caused by average re-referencing. Epochs time-locked to a STD stimulus presentation that directly followed a DEV stimulus were excluded. Moreover, epochs containing notable non-stereotyped artifacts were removed using the criteria reported in Figure 2. For each participant, remaining epochs were concatenated and then decomposed by adaptive mixture ICA (AMICA) (Palmer et al., 2008). The mean numbers (SD and ranges) of the epochs and channels used for ICA decomposition are reported in Figure 2. ICA decomposition identified a total of 1121 IC processes in the adult group (mean, 62.3 ICs/subject), and 1919 ICs in the infant group (mean, 56.4 ICs/subject).

### Independent Component Localization

For each IC, the location in a template brain of the best-fitting single equivalent dipole or bilaterally constrained equivalent dipole pair was estimated. For infant data, this process was performed using an age-appropriate four-layer electrical forward problem head model template generated by the NFT toolbox (Akalin Acar and Makeig, 2010; Piazza et al., 2016b). Adult IC equivalent dipole modeling was performed using the EEGLAB plug-in DIPFIT based on a three-shell boundary element method (BEM) head model built on a Montreal Neurological Institute (MNI) template. In both cases, the inverse problem was solved using functions from the fieldtrip toolbox (Oostenveld et al., 2011). These equivalent dipole locations were assumed to represent spatially coherent activity across a cortical patch; simulations show that the equivalent dipole location is typically quite near to the center of such a source patch (Akalin Acar and Makeig, 2013).

Independent component scalp maps that showed a bilaterally near-symmetrical projection pattern were identified and modeled with two bi-symmetrically location-constrained (but orientation unconstrained) equivalent dipoles using the *fitTwoDipoles* EEGLAB plug-in (Piazza et al., 2016a). The plug-in performed an automatic selection of bilateral ICs by identifying IC scalp maps that appear to be bilaterally symmetrical; the following plug-in parameters were used: Symmetry region, “Large Rectangular”; Symmetric peak detection threshold, 35%. These parameters were chosen in order to maximize the accuracy of the bilateral IC selection algorithm, preserving both specificity and sensitivity (Piazza et al., 2016a). The constraint for equivalent dipole pairs to be bilaterally location-symmetric minimized the chance of multiple local minima in the equivalent dipole model solution space. The selection of ICs classified as dual-dipolar was then optimized by an experienced experimenter (the first author) by visual inspection of the scalp maps, as previously suggested (Piazza et al., 2016a). For both groups, the percentage of ICs so re-classified was lower than 10%.

### Independent Component Selection and Clustering

Independent component analysis decomposition of EEG data returns ICs accounting for eye movement, scalp muscle, line noise, electrocardiographic (ECG), and single-channel activities, as well as a “noise subspace” of ICs with small scalp projections and not further characterizable as to source or function. It is necessary, therefore, to distinguish ICs compatible with an origin within the brain to focus further analysis on brain activity contained in the recorded scalp signals. Next, to support group analysis, one needs to cluster similar ICs across participants.

Here we accomplished this using the k-means clustering approach implemented in EEGLAB. For each group of subjects, an EEGLAB STUDY data structure [1 × 3 design: 1-group × 3-stimulus types (STD, FDEV, DDEV)] was created and two consecutive clustering procedures were applied (Piazza et al., 2016b). The first clustering procedure was performed to identify and then reject from further consideration ICs not clearly compatible with a cortical effective source. Only ICs whose equivalent dipole model was estimated to be inside the brain were used in the analysis; thereby, 1596 (46.9 ICs/subject) and 786 (43.7 ICs/subject) ICs were retained for the infants and adults, respectively. In this preliminary clustering procedure, we decided to use a large number of clusters to increase the probability of isolating artifact ICs from brain ICs. Thus, 60 clusters were created for the infant ICs and 30 clusters for the adult ICs. The following IC measures were used in the clustering to define the IC distance metric: mean log power spectra (frequency range: 1–40 Hz; relative weighting: 5; infant dimension: 30, adult dimension: 15) and scalp maps (relative weighting: 3; infant dimension: 30; adult dimension: 15). Successively, clusters clearly related to non-EEG sources were rejected and ICs whose equivalent dipole model, when projected to the scalp, had high residual variance (rv) from the actual IC scalp map were excluded (rv threshold for rejection was set to 25% for infant data and 20% for adult data; these values were empirically identified as detailed in Piazza et al. (2016b).

A second clustering procedure was then performed on the remaining putative “brain” ICs – 824 ICs for infants (24.2 ICs/subject) and 391 ICs for adults (21.7 ICs/subject). The following measures were used: equivalent dipole location (relative weighting: 10; dimension: 3) and ERP responses to STD and DEV stimuli in the 700 ms following stimulus onset (concatenated; relative weighting: 5; infant dimension: 17, adult dimension: 9). The number of clusters was selected based on previous studies (e.g., Rissling et al., 2014) that suggest limiting the number of clusters to 20 or less to enhance the chance for each cluster to include at least one IC from each subject. For the infant group, we created 20 clusters, as in Piazza et al. (2016b). On average, each cluster contained 39.1 ICs (SD = 12.4) with 1.2 (SD = 0.2) ICs/subject.

Since the adult group had fewer subjects and, thereby, ICs, we reduced the number of clusters. Eleven clusters were created and on average 34.1 ICs were contained in each cluster with 1.8 (SD = 0.7) ICs/subject. Clusters with a sizeable proportion (≥20%) of bilateral ICs modeled by a symmetric dipole pair were then identified. For each bilateral IC in these clusters, the ratio between the magnitudes of the left and right dipole moments was computed.

For each group, the five IC clusters contributing most strongly to the grand-mean scalp ERPs were identified based on the cluster-mean percent variance accounted for (*pvaf*) in the scalp channel ERP across the 0–700 ms time window (Piazza et al., 2016b).

Finally, IC cluster centroids were localized in functional brain space using the Talairach Applet (Lancaster et al., 2000). For infant data, a translation from the infant template MRI coordinates to the MNI standard brain was first performed (Piazza et al., 2016b).

### Statistical Analysis

The statistical validity of the difference between groups in the number of bilateral ICs per subject was assessed by independent sample *t*-test. Only the ICs used in the final clustering procedure were used in the statistical analysis. A *post hoc* power analysis was also performed using the GPower (3.1.9.4) application to verify if the sample size was adequate to detect the identified effect by inferential statistics.

Hemispheric dominance effects were assessed in the clusters with at least the 20% of bilateral ICs by performing independent Wilcoxon tests on left and right dipole moment magnitudes. All statistical analyses were conducted using IBM SPSS (“Statistics 21”).

## Results

The aim of our study was to investigate the presence of bilateral IC effective source patches in both adults and infants, to verify if bilaterally symmetric synchronous activity between the two hemispheres could be considered a special or more prominent dynamic feature of the infant brain.

**FIGURE 3:**
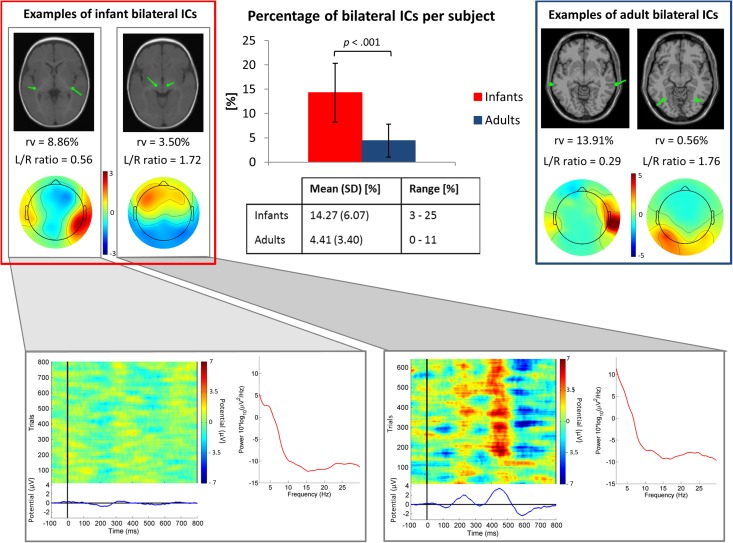
*Top center.* Bar graph showing the mean percentage of ICs judged to be bilateral per subject. The group difference was significant (*p* < 0.001 by independent sample *t*-test). Error bars present standard deviations. In the table, mean values, standard deviations (SD) and ranges (min-max) of the percentage of bilateral ICs for each group are shown. Top left and right boxes. Example bilateral infant (left) and adult (right) IC scalp maps and dipole models. *Bottom.* Power spectra, ERP-image plots, and mean ERPs for the two example infant bilateral ICs. Here all trials were included in order of presentation, independent of stimulus type.

Our results showed that in the infant group 13.8% (*N* = 114 ICs) of the 824 clustered “brain” ICs had a sufficiently marked bilateral pattern to be modeled using two symmetric dipoles (3.3 ICs/subject). Whereas, in the adult group only 4.3% (*N* = 17) of the 391 clustered “brain” ICs were modeled with a position-symmetric bilateral equivalent dipole model (0.9 ICs/subject). The percentage of bilateral ICs per subject differed significantly between infants and adults, *t*(50) = -6.36, *p* < 0.001 (Figure 3). The *post hoc* power analysis gave a statistical power of 0.99. In Figure 3, power spectra and evoked responses for two example infant bilateral ICs are shown. This figure suggests that the synchronous activity identified by ICA decomposition was related both to ongoing, non-stimulus locked EEG activity (Figure 3, bottom left) and to the auditory stimulus-locked response activity (Figure 3, bottom right).

**FIGURE 4:**
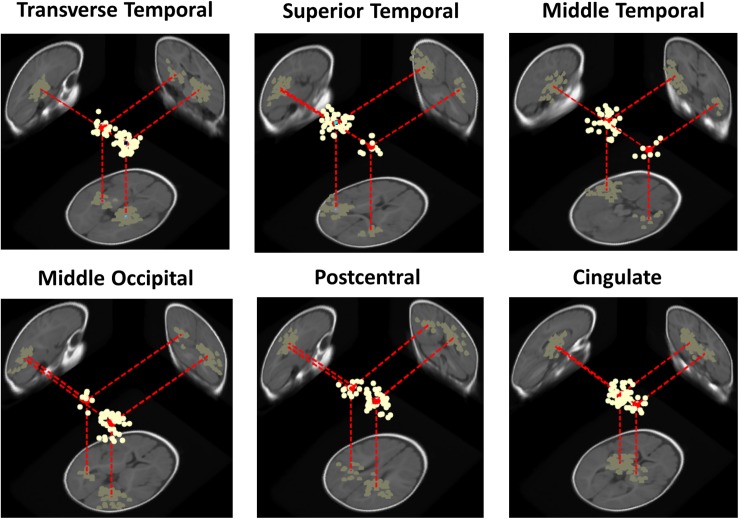
Three-dimensional equivalent dipole models of ICs in the bilateral infant clusters. Dipole projections are shown in the sagittal, coronal, and transverse planes of the MNI infant template brain model. Cluster dipole centroids are shown in red.

At the cluster level, for the adult group no IC clusters met the 20% “bilaterality” threshold, whereas for the infant group, in 30% (*N* = 6 of 20) of the IC clusters at least the 20% of ICs were modeled using two equivalent dipoles. Figure 4 shows template brain locations of the equivalent dipole models for ICs in these six clusters. Note that simulations have shown that, because of unaccountable individual variations in skull conductance, the radial depth of the imputed dipole locations can be less well estimated than the solid angle from the brain center to the equivalent dipole source location (Akalin Acar and Makeig, 2013). This may account for the implausibly deep dipole locations for some ICs.

**FIGURE 5:**
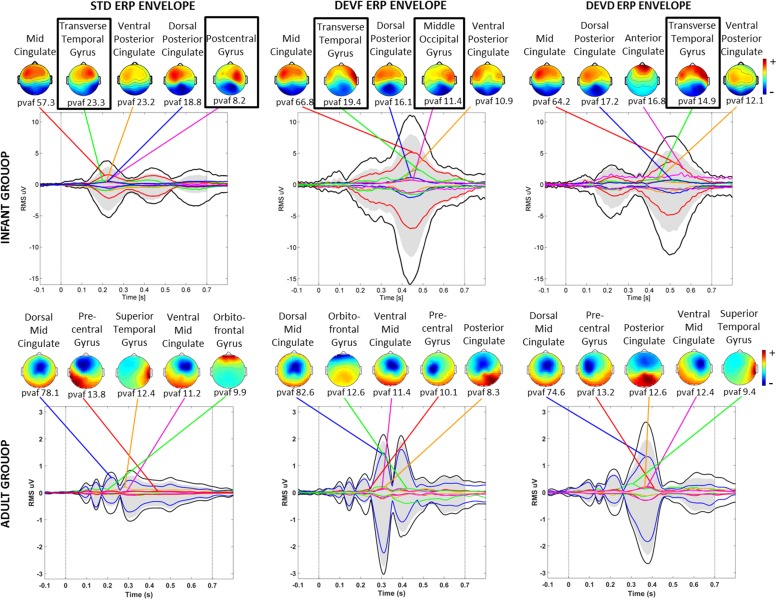
Scalp topographies of the summed ERP projections of the most strongly contributing IC source clusters. From left to right graphs related to each stimulus type (STD, FDEV, and DDEV) are displayed. Top: infant group; bottom: adult group. For each cluster, the percent variance accounted for (pvaf) in the scalp channel ERP is shown below its scalp map. Black traces in each graph show the envelope (the most positive and negative single-channel values at each latency) of the grand-average scalp channel ERP. Upper and lower edges of the gray areas represent the envelope of the summed contributions of the five contributing clusters. Colored traces show the envelopes of the summed cluster-IC scalp projections. Black boxes identified bilateral clusters.

Three of the bilateral clusters were located in the temporal lobe, in or near Transverse Temporal Gyrus, Middle Temporal Gyrus, and Superior Temporal Gyrus, thus close to the auditory cortex. The other bilateral clusters were located in the parietal, occipital and limbic lobes, specifically in or near Post-Central Gyrus, Middle Occipital Gyrus, and Cingulate Gyrus. The Transverse Temporal Gyrus, Post-Central Gyrus, and Middle Occipital Gyrus clusters were among the source clusters accounting for the largest part of the scalp grand-mean ERPs, as shown in Piazza et al. (2016b) and in Figure 5, where the scalp topographies of the summed ERP projections of the most strongly contributing IC source clusters for each stimulus type are shown.

**FIGURE 6:**
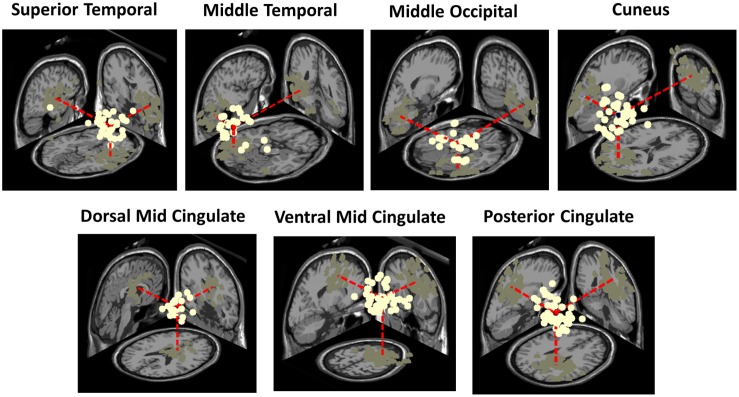
Three-dimensional equivalent dipole model locations of ICs contributing to the adult clusters having most similar centroid locations to the bilateral clusters identified in the infant group. Dipole projections are shown in the sagittal, coronal, and transverse planes of the MNI template brain. Cluster dipole centroids are shown in red.

Clusters located in similar positions were identified in the adult group, but these were characterized by unilateral component activities (Figure 6). Specifically, we found two adult data source clusters in the temporal lobe, in or near Right Superior Temporal Gyrus and Left Middle Temporal Gyrus; two source clusters in the occipital lobes, in or near the Right Middle Occipital Gyrus and the Cuneus and three source clusters in the limbic lobe, in or near the Cingulate Gyrus. No clusters were centered in the parietal lobe. Four of these IC clusters are among the clusters contributing most strongly to the grand-mean scalp ERPs for the adult group (Figure 5).

In Table 1, we report the total number of ICs, the percentage of bilateral ICs, and the number of subjects contributing to each of the bilateral clusters for the infant group and to each similarly locate cluster in the adult group.

**TABLE 1:** The number of independent components (ICs), percentage of bilateral ICs, and number of subjects contributing to each of the six bilateral clusters for the infant group (upper panel) and to each similarly located cluster in the adult group (lower panel).

Source cluster	# ICs	% Bilateral	# Subjects
**Infant group**			
Transverse Temporal Gyrus	51	37	29
Middle Temporal Gyrus	38	21	24
Superior Temporal Gyrus	39	31	22
Post-Central Gyrus	37	35	24
Middle Occipital Gyrus	38	21	24
Cingulate Gyrus	45	27	24
**Adult group**			
Right Superior Temporal Gyrus	35	2	15
Left Middle Temporal Gyrus	29	7	16
Right Middle Occipital Gyrus	26	0	13
Cuneus	38	8	15
Dorsal Mid Cingulate	17	6	16
Ventral Mid Cingulate	44	7	15
Posterior Cingulate	53	2	17

In Table 2, we report, for all these clusters, the number of single-dipole ICs with equivalent dipole locations in the left or the right hemispheres respectively. For bilateral ICs in the infant clusters, we report the median (and interquartile range) of the dipole moment left/right magnitude ratios. Here a left/right ratio below 1 corresponds to a stronger scalp projection of right hemisphere dipole than of the left hemisphere dipole, above 1 to the opposite. Each bilateral cluster included more unilateral ICs in one or the other hemisphere. Moreover, the left/right dipole moment ratios for the dual-dipolar ICs matched the left/right distribution of unilateral ICs in the same clusters.

**TABLE 2:** The number of the left and right unilateral independent components (ICs) and the median (and interquartile range) ratios between the left and right dipole moment magnitudes for the dual-dipolar ICs in each infant cluster with > 20% bilateral ICs.

Source cluster	# Unilateral left	# Unilateral right	L/R ratio	Wilcoxon test	
				Z	*p*
**Infant group**					
Transverse Temporal Gyrus	0	32	0.48 (0.28)	3.823	<0.001
Middle Temporal Gyrus	30	0	2.33 (0.94)	−2.240	0.025
Superior Temporal Gyrus	27	0	2.40 (1.04)	−3.059	0.002
Postcentral Gyrus	0	24	0.44 (0.33)	3.180	0.001
Middle Occipital Gyrus	0	30	0.31 (0.10)	2.521	0.012
Cingulate Gyrus	31	2	2.52 (1.59)	−3.059	0.002
**Adult group**					
Right Superior Temporal Gyrus	0	34			
Left Middle Temporal Gyrus	27	0			
Right Middle Occipital Gyrus	0	26			
Cuneus	23	12			
Dorsal Mid Cingulate	3	13			
Ventral Mid Cingulate	7	34			
Posterior Cingulate	10	42			

*Statistics and *p*-values for the Wilcoxon test performed on left and right dipole moment magnitudes (upper panel). The number of the left and right unilateral ICs contributing to each similarly located cluster in the adult group (lower panel). No L/R ratio was reported for the adult clusters due to the low number of bilateral ICs in each cluster.*

## Discussion

To our knowledge, this is the first study that compares ICA decompositions of infant and adult EEG data collected in the same paradigm. ICA decomposition is a new means to characterize the development of functional brain organization in early life. Based on previous experience with adult data, we expected them to yield a smaller number of bilateral IC effective brain sources than infants, in line with expectations that increases in brain specialization during early development might reduce the extent of temporally and functionally synchronous local field source activities in left and right cortical areas anatomically and functionally linked by commissural fibers.

Cortical architecture is highly weighted toward short-range connections, wholly so for inhibitory cell networks. This suggests that brain-based (non-artifact) IC effective sources of scalp EEG should be associated with processes arising from stationary, locally coherent (or partially coherent) field activities in physically distinct, cortical source areas of unknown size (possibly estimable using an electrical head model built on the subject-specific head MR image, see Akalin Acar et al., 2016). This concept can account for the many brain source IC scalp maps that match the topographic projection pattern of a single oriented equivalent dipole (Delorme et al., 2012). Cortical source signals generated in more than one cortical patch might be (fully or partially) synchronized if their source patches are bidirectionally connected neurophysiologically, e.g., by corpus callosum or other white matter tract, and/or possibly when they respond to external stimuli with near identical time courses, e.g., as regulated by respective thalamic and/or cortical inputs. In these cases, ICA decomposition might return a component summing the scalp projections of the two source patches whose activities are thereby partially synchronized. The IC scalp map for such a component should be well modeled using two equivalent dipoles symmetrically (or near symmetrically) located in the left and right hemispheres (with possibly dissimilar dipole orientations because of local differences in cortical topography). Thus, ICA decomposition can not only identify independent (or nearly independent) processes contributing temporally distinct information to the scalp recording, but can also give information about brain functional organization. Specifically, it can reveal functional connectivity associated with coordinated synchronous activity (Friston, 2011). For this reason, ICA decomposition may be particularly promising for the study of functional organization in the infant brain (Piazza et al., 2016b).

Previously (Piazza et al., 2016b), we noted that ICA decompositions of these infant EEG data exhibited a relatively large number of bilateral ICs. The results of the present work support the hypothesis that this phenomenon is characteristic of the infant brain, since here EEG data from a group of healthy young adults, tested under parallel circumstances, produced nearly the same number of labeled “brain” ICs, but a significantly lower number of these could be identified as dual-dipolar. Moreover, our results (Figure 3) suggest that the identified bilateral activity was related not only to responses to auditory stimulation but was a feature as well of ongoing EEG activity. Indeed, three of the infant “bilateral” clusters were not among those strongly contributing to the scalp ERPs, as reported in Piazza et al. (2016b) and in Figure 5. This further supports the idea that synchronous activity within and between (most likely) directly coupled bilateral source patches is characteristic of the overall functioning of the infant brain.

Although some structural brain asymmetries are clearly present in the first stages of life, the development of functional asymmetry is more debated. Some authors (e.g., Lenneberg, 1967) have postulated that each brain hemisphere might have the same potentiality at the beginning of life. The fact that early lesions are followed by better behavioral recovery compared to adults supports this hypothesis. However, brain-imaging studies in infants have contradicted this hypothesis by revealing asymmetrical hemodynamic and metabolic response patterns similar to those identified in adults in response to auditory and visual stimuli (e.g., Dehaene-Lambertz et al., 2002; Tzourio-Mazoyer et al., 2002). Both bilateral and lateralized activations have been reported in infant fNIRS and EEG experiments (e.g., Telkemeyer et al., 2009, 2011; Minagawa-Kawai et al., 2011; Ortiz-Mantilla et al., 2012).

Our results suggest the presence, in the infant data, of synchronous bilateral component activities that are often stronger in one hemisphere than in the other. This characteristic was present in all the bilateral clusters identified. For example, the Transverse Temporal Gyrus cluster (Figure 4), with the highest percentage of bilateral ICs and highest subject penetration, appears to present a slight right lateralization, as the 32 unilateral IC equivalent dipoles within this cluster were all in the right cortex and, accordingly, the right-hemisphere dipole moments of the 19 dual-dipolar ICs were on average more than twice as strong as their left-hemisphere dipole moments (*p* < 0.001). The absence of bilateral clusters in the adult data supports the idea that the stronger lateralization in adults of both functional brain imaging activity and of IC EEG effective source distributions are consequences of brain maturation.

Finally, our results demonstrate that the immature brain features locally synchronous electrical activity patterns in bilaterally coupled cortical areas. Interestingly, hemispheric *asymmetry reduction* in older adults (HAROLD) (Cabeza et al., 2002; Reuter-Lorenz and Park, 2010), also referred to as de-differentiation, indicates deviation from hemispheric specialization of function, and is considered to possibly represent a compensatory mechanism to counteract functional decline during aging. As senescence has thus been associated with bilateral hemisphere recruitment, our results here suggest that infancy and senescence may produce an inverse U-shaped curve in functional brain bilaterality with age, indicating the brain’s general principle of trading efficiency for processing power to regulate performance in the early and late stages of life.

It should be noted that our results rest on the adequacy of the identification here of the dual-dipolar ICs. To make the selection as objective as possible, we use the automated selection algorithm implemented in the *fitTwoDipoles* plug-in. The parameters we used for bilateral IC selection were optimized in terms of both specificity and sensitivity (Piazza et al., 2016a), and the parameters used in the plug-in were the same for both groups, minimizing the chance of identification bias. Nevertheless, positive and/or negative selection errors might have occurred. For example, ICs whose scalp maps were faintly bilateral (i.e., strongly left or right dominant) might have been misclassified as single-dipole. While this might have minimized the identified numbers of dual-dipole ICs in the adult group, the fact would remain that our results using the (potentially less sensitive) *fitTwoDipoles* threshold for detecting dual-dipolarity here demonstrate that, in adults, remaining bilaterality in maximally independent EEG effective source processes is at best faint relative to that exhibited in infant EEG.

To re-verify the results of the *fitTwoDipoles* selection, visual re-inspection of the classification results was performed, as suggested in Piazza et al. (2016a), to identify misclassified ICs. The correspondence between the automatic selection and the visual check was at least 90% in both groups, thus underlying the reliability of the automatic selection and the low influence of subjective visual inspection on the results.

Some concerns might emerge in relation to the different amounts of EEG data, in terms of numbers of retained channels and data epochs, used for ICA decomposition in the two groups (Figure 2), as well as in relation to the different amplitude threshold used for abnormal epoch detection (Figure 2). In the infant group, we used a higher amplitude threshold than for adults, since infant EEG signals are characterized by greater amplitudes (Bell and Wolfe, 2008). Nevertheless, in the infant group, more channels and epochs were discarded than for adults. This is because of the higher number of artifacts (mainly movement-related) included in the infant data. However, for each subject in both groups, the number of data points in relation to the number of channels was sufficient for performing ICA decomposition. Specifically, given N channels the number of data points decomposed were at least equal to kN^2^ with k ≥ 20 (Onton et al., 2006). This supports the reliability of the ICA decomposition on which the main result of the present study is based, i.e., the identification of more bilateral IC effective source patches in infants then in adults. This result is also unaffected by the different head models used for the two groups of subjects that eventually could only interfere on the precise location of the dipoles. Moreover, it should be noted that the clustering method we used presents some limitations, first of all the fact that it depends on the measure subset used as clustering criteria and requires defining *a priori* the number of clusters. Thus, different clustering solutions can be produced. This might have some effect on the bilateral clusters identified. However, the percentage of bilateral ICs in the adult clusters is always far below the 20% value selected as threshold to define a cluster as bilateral. Thus, we believe that it is strongly improbable that different clustering solutions can reveal bilateral clusters in the adult group.

Finally, readers used to measuring ERPs computed from EEG data might note that although we here applied ICA decomposition to sets of concatenated 900-ms whole data epochs time locked to auditory stimulus presentations, the amount of whole data variance accounted for by the trial-average ERPs was, as typical, quite small. Specifically, the ERP trace variance computed across the 0–700 ms time window was on average 0.55 μV^2^ in the adult group and 2.62 μV^2^ in the infant group. The brain-based component processes identified by ICA decomposition accounted for much more of the ongoing EEG data in these epochs than the evoked-response alone. Indeed, the average *pvaf* by the clustered brain IC contributions to the recorded scalp EEG data was 61.2% in the adult group and 59.4% in the infant group. Thus, the group differences we report here were likely not linked to differences in bilaterality of the auditory ERPs for these epochs.

To our knowledge, this is the first study to compare infant and adult brain IC sources of high-density EEG data. Taking into account the physiological interpretation of brain-based ICs as physically distinct sources that typically also exhibit functional independence, the identified difference in the proportion of bilateral brain IC sources in infants and adults provides a valuable approach to investigating the functional organization of the infant and developing brain, both typical and abnormal, potentially leading to improvements in diagnosis and rehabilitation of neurodevelopmental disorders. Thus, future studies with larger sample size, possibly longitudinal and involving clinical populations are encouraged.

## Data Availability Statement

The datasets generated for this study are available on request to the corresponding author.

## Ethics Statement

The studies involving human participants were reviewed and approved by the Ethics Committee of the Scientific Institute IRCCS E. Medea (Bosisio Parini, LC, Italy). Written informed consent to participate in this study was provided by the participants or by their legal guardian/next of kin.

## Author Contributions

CP, CC, MMo, GR, and SM designed the study. CP, CC, and VR run the experiment and collected the data. CP analyzed the data. CP, MMi, and SM interpreted the results. CP drafted the manuscript. All authors edited and revised the manuscript.

## Conflict of Interest

The authors declare that the research was conducted in the absence of any commercial or financial relationships that could be construed as a potential conflict of interest.
